# Supplementary material for: COVID-19 Breakthrough Infections and Transmission Risk: Real-World Data Analyses from Germany’s Largest Public Health Department (Cologne)
Source: Vaccines (Basel). 2021 Nov 2;9(11):1267. doi: 10.3390/vaccines9111267 (PMC8624814; doi:10.3390/vaccines9111267)
Supplement: Supplementary file 1 [file vaccines-09-01267-s001.zip › vaccines-1430458-supplementary.pdf]

Supplemental material Table S1

	Regression coefficient (*B*)	Std. error	Sig.	OR	95% confidence interval	
					Lower limit	***Upper limit***
Age	0.001	0.003	0.754	1.00	1.00	1.01
Gender (female = 1; male = 2)	0.088	0.127	0.486	1.09	0.85	1.40
Vaccination status of CP (unvaccinated = 1; fully vaccinated = 0)	0.234	0.172	0.174	1.26	0.90	1.77
Vaccination status of source case (CP of VG = 1; CP of CG = 2)	−1.584	0.144	<0.001	0.21	0.16	0.27
